# Predictors of Success of Pharyngeal Surgery in the Treatment of Obstructive Sleep Apnea: A Narrative Review

**DOI:** 10.3390/jcm12216773

**Published:** 2023-10-26

**Authors:** Heloisa dos Santos Sobreira Nunes, Joana Vaz de Castro, Valentin Favier, Florent Carsuzaa, Marina He Ryi Kim, Felipe Ahumada Mira, Giuseppe Meccariello, Claudio Vicini, Andrea De Vito, Jerome R. Lechien, Carlos Chiesa Estomba, Antonino Maniaci, Giannicola Iannella, Giovanni Cammaroto

**Affiliations:** 1 ENT and Sleep Medicine Department, Nucleus of Otolaryngology, Head and Neck Surgery and Sleep Medicine of São Paulo, São Paulo 04090-010, Brazil; 2 Young Otolaryngologists-International Federations of Oto-Rhinolaryngological Societies (YO-IFOS), 75000 Paris, Francevalentin_favier@hotmail.com (V.F.); felipe.ahumada.m@gmail.com (F.A.M.); tnmaniaci29@gmail.com (A.M.);; 3 ENT Department, Armed Forces Hospital, 1649-026 Lisbon, Portugal; 4 ENT Department, University Hospital of Montpellier, 34080 Montpellier, France; 5 ENT Department, University Hospital of Poitiers, 86000 Poitiers, France; 6 ENT Department, Hospital of Linares, Linares 3582259, Chile; 7 Head and Neck Department, ENT & Oral Surgery Unity, G.B. Morgagni, L. Pierantoni Hospital, 47100 Forlì, Italy; 8 Division of Laryngology and Broncho-Esophagology, Department of Otolaryngology and Head and Neck Surgery, EpiCURA Hospital, UMONS Research Institute for Health Sciences and Technology, University of Mons, 7000 Mons, Belgium; 9 Department of Otorhinolaryngology, Donostia University Hospital, Biodonostia Research Institute, Osakidetza, 20014 San Sebastian, Spain; 10 Department of Medical and Surgical Sciences and Advanced Technologies “GF Ingrassia”, ENT Section, University of Catania, Piazza Università 2, 95100 Catania, Italy; 11 Department of ‘Organi di Senso’, University “Sapienza”, Viale dell’Università 33, 00185 Rome, Italy

**Keywords:** obstructive sleep apnea, soft palate, drug-induced sleep endoscopy, phenotypes, pharyngoplasty, sleep surgery

## Abstract

(1) Background: This narrative review aims to explore the predictors of success for pharyngeal surgery in the treatment of obstructive sleep apnea (OSA). An extensive literature search was conducted, identifying relevant studies published up to June 2023, utilizing various databases and key search terms related to OSA, surgical interventions, and predictors of success. The review encompasses both retrospective and prospective studies, case series, and cohort studies to provide a broad understanding of the topic; (2) Methods: Review of English scientific literature on phenotypes of OSA related to predictors of success of pharyngeal surgery; (3) Results: Of 75 articles, 21 were included, in these the following were determined to be factors for surgical success: body mass index (BMI) (8 articles), apnea/hypopnea index (AHI) (8 articles), cephalometry (8 articles), palatine tonsil size (7 articles), Modified Mallampati score (2 articles), genioglossus electromyography (2 articles), Friedman score or upper airway anatomy (3 articles), nasopharyngolaryngoscopy (2 articles), drug-induced sleep endoscopy (DISE) (1 article), oral cavity anatomy (1 article) and oxygen desaturation index (ODI) (1 article); (4) Conclusions: The lack of standardized protocols for the indication of pharyngeal surgery is a reality, however identifying known predictors of surgical success may facilitate homogenizing indications.

## 1. Introduction

Obstructive sleep apnea (OSA) is a nocturnal breathing disorder linked to the abnormally frequent occurrence of respiratory pauses. This occurs due to a combination of mechanisms such as upper airway collapse, decreased muscle responsiveness, decreased arousal threshold, and low ventilatory drive (loop gain). Critical closing pressure (Pcrit) is the gold standard measure for the degree of collapsibility of the pharyngeal airway, and this is particularly important in OSA, where 70% of the upper airway [1] collapse occurs at the pharyngeal level. Disturbances in gas exchange lead to oxygen desaturation, hypercapnia, and consequentially increased arousals and hence sleep fragmentation and a pro-inflammatory state. This leads to OSA-related symptoms complaints and known comorbidities, such as cardiovascular, metabolic, and neurocognitive disorders [2,3]. OSA is present in approximatively 9 to 38% of adults, increases with age, and is more frequent in men, however, after menopause this gap decreases [4]. A major cause of excessive sleepiness is a reduced quality of life, impaired work performance, and an increased motor vehicle crash risk [5,6,7], given its increasing prevalence and its close relationship to numerous morbidities, OSA is nowadays, a major public health problem [8].

The treatment choice should be based on the pathogenesis of the airway collapse, and the severity of OSA and must relieve symptomatic and disease burden. This systematically includes behavioral measures such as abstinence from alcohol and sedatives, regular physical exercise, and weight loss (>5–10% of total weight). Although the most effective method of maintaining the upper airway open during sleep remains the administration of positive air pressure (PAP), this has limited effectiveness mainly due to low patient compliance and in those that comply, end-of-night removal [9,10]. Numerous proposals for oral appliances have been developed to treat OSA. Mandibular advancing devices (MADs) are emerging as the most extensively researched among them. These mechanisms are specifically engineered to maintain the mandible and tongue in an advanced position throughout the night, with the added aim of enhancing the vertical dimension [11]. Regarding OSA treatment, pharyngeal surgery is a modality that improves OSA without relying on a device, and therefore efficacy and effectiveness are one and the same, as there is no need for compliance. In selected patients, upper airway surgery is a powerful tool that may return upper airway patency and even delay the natural OSA progression with age. Several surgical methods have been introduced to eliminate the mechanism of upper airway collapse among which include, traditional soft palate surgery and, more recently, a combination of velopharyngeal and pharyngeal lateral wall surgeries, which we consider as pharyngoplasties. Uvulopalatopharyngoplasty (UPPP), initially described by Fujita et al. in 1981 was the first surgical procedure of the soft palate level designed to treat OSA, and included excision of the tonsils, trimming and relocating the tonsillar pillars, uvulectomy and mucosal closure of the soft palate [12]. Although effective in reducing symptoms, this surgery was criticized for its severe postoperative pain, complications, and success limited to selected patients [13]. In a 2019 analysis of surgical predictors using drug-induced sleep endoscopy (DISE), the predictors of decreased surgical success (analyzing all types of pharyngoplasties or multilevel surgeries) were velopharyngeal concentric, oropharyngeal lateral wall and tongue base collapse. In articles treating multilevel collapse with multilevel surgery; oropharyngeal lateral wall, tongue base, and epiglottic collapse no longer were predictors of surgical failure, as these would be addressed in the same procedure. Predictors of success were tonsillar hypertrophy (grade 3 or 4), anteroposterior velopharyngeal, partial tongue base, or epiglottic collapse [14]. To understand who best responds to upper airway surgery, it is of the utmost importance to classify the type of collapse, patient phenotype, and type of surgery performed. This review focuses on the most common location for OSA collapse, and predictors of success or failure.

Over the past 50 years, many surgical interventions targeting the pharynx have been described and shown promise in improving the symptoms and overall outcomes of OSA patients [15]. However, the effectiveness of each of these techniques is related to individual factors that must be identified before offering the best treatment to the patient. This narrative review aimed to explore the predictive factors of success for pharyngeal surgery in the treatment of OSA.

## 2. Materials and Methods

A comprehensive literature search was conducted, identifying relevant studies published up to June 2023, utilizing various databases and key search terms related to OSA, surgical interventions, and predictors of success. This review encompasses both retrospective and prospective studies, case series, and cohort studies to provide a broad understanding of the topic.

A review of the literature, of articles written in English, on the phenotypes of OSA was performed using PubMed, Lilacs, Cochrane, and ScIELO electronic databases. Three searches using 1/pharyngoplasty sleep apnea, 2/soft palate surgery, and 3/predictors of success as keyword clusters were performed and were combined with the use of the “AND” function to better select the research. Each article included in the study met the following inclusion criteria:

(1) Age >18 years
(2) Velopharyngeal or lateral pharyngeal wall surgical treatment of OSAS patients,
(3) Predictors of success related to phenotypes: anatomical, low arousal threshold, ventilatory instability, and poor muscle response
(4) Pre-operative evaluations: DISE, body mass index (BMI), apnea-hypopnea index (AHI) with all levels of PSG, lateral wall space, positional OSA, Friedman classification, cephalometry, nasopharyngolaryngoscopy and nasopharyngeal tube

Articles that were not in accordance with the inclusion criteria were excluded. The subsequent criteria were applied with the aim of excluding inappropriate studies:

Surgical technique reports without significant outcome data, articles consisting of meta-analyses or literature reviews on surgical procedures, and articles describing animal or cadaveric samples. In order to further reduce the risk of an incomplete literature analysis, a manual search through the bibliography of the included articles was carried out.

The pathophysiology of OSA in children is generally attributed to either adenotonsillar hypertrophy and/or obesity, except in rare situations such as syndromes, laryngomalacia, and neuromuscular or craniofacial disorders, amongst other conditions. In most cases, adenotonsillectomy is offered with a high success rate for the treatment of OSA, and there are already guidelines indicating ideal candidates for surgery, therefore, the focus of this review is adults.

As children are not a population that commonly undergoes pharyngeal surgery other than adenotonsillectomy, the focus of this review is on adults.

The rate of success after surgery was considered to ensure that articles are aligned with the topic “predictors of success”, but it was not an inclusion criterion since there are articles without a success rate described.

## 3. Results

**Figure 1:**
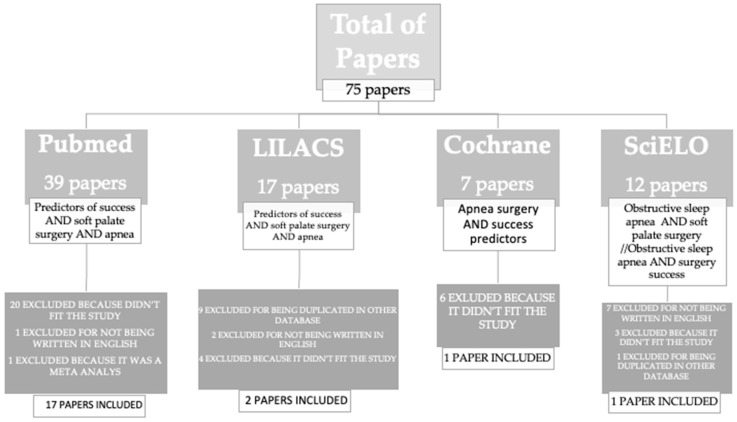
Articles selection criteria for the review.

**Figure 2:**
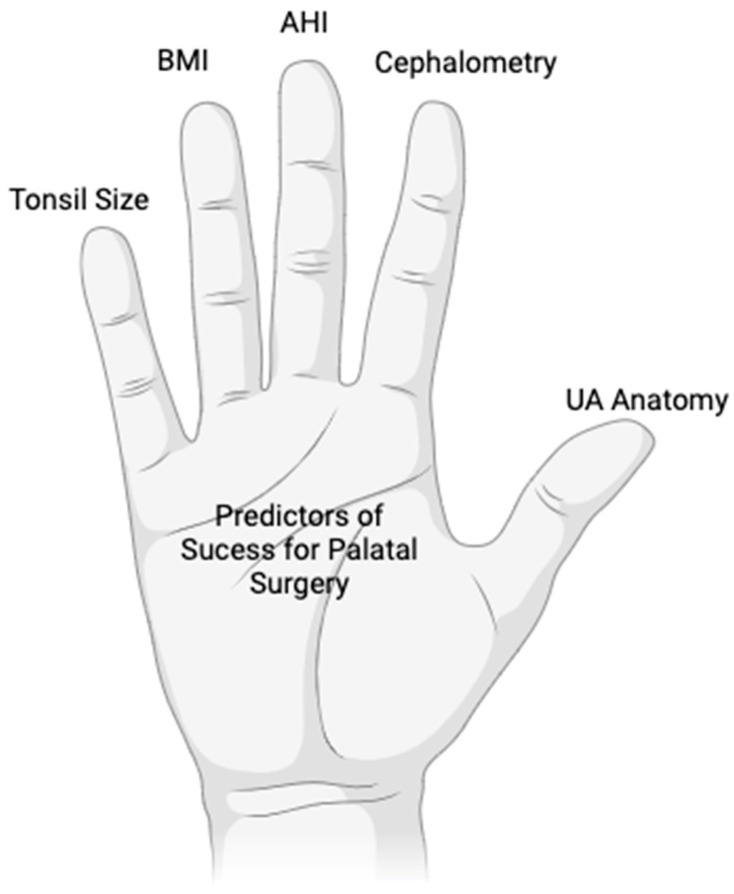
Main predictors for palatal surgery success in the palm of the hand.

**Figure 3:**
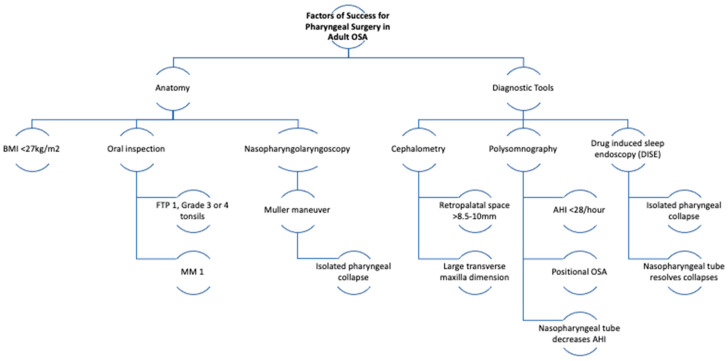
Predictors of pharyngeal success in relation to anatomic or diagnostic data.

From 1988 Until July 2023, we found a total of 75 articles. 39 on Pubmed, 12 on SciELO, 17 on LILACS, and 7 on Cochrane. Duplicated articles, as well as studies related to multilevel surgeries without a soft palate approach, articles not addressing palatal surgery, non-phenotype approach, and inconclusive were excluded. A total of 20 articles were included, 7 articles addressed BMI as a predictor, 8 articles suggested AHI, 8 articles for cephalometry, 7 for tonsil size, 2 for Modified Mallampati score, 2 for genioglossus electromyography, 3 for Friedman score or upper airway anatomy, 2 for nasopharyngolaryngoscopy, 1 for DISE, 1 for oral cavity anatomy and 1 suggesting that oxygen desaturation index (ODI) is a predictor for success for pharyngeal surgery. The results and criteria are described in Figure 1 and the selected articles are described in Table 1. We group the main predictors in Figure 2 as a mnemonic way to remember and present. The diagram with the summary of predictors is in Figure 3.

## 4. Discussion

Obstructive sleep apnea syndrome (OSAS) is characterized by recurrent episodes of airway obstruction during sleep, which is usually associated with sleep fragmentation and oxygen desaturation. The diagnostic golden standard for OSA is polysomnography (PSG), which needs to fulfill the following criteria: at least five episodes of apnea or hypopnea per hour, associated with OSA-related symptoms and comorbidities, of which the hallmark symptom is daytime sleepiness. OSA patients tend to have anatomic characteristics that potentiate upper airway collapse, however, not all cases should be solved with surgical intervention, but may be treated with other approaches like PAP, MAD, myofunctional therapy, and positional therapy.

Since 1981 many velopharyngeal surgical techniques have arisen, mainly to respect the anatomy and physiology of the pharynx, and address more than anteroposterior pharyngeal collapse, but also lateral wall collapse [12,15]. The tendency currently is to choose the technique that the surgeon is most familiar with, that addresses the patient’s anatomy and physiology (collapses observed during sleep, as well as awake), that is based more on relocation and preservation of tissue, rather than resection.

Various findings have been associated with the severity of OSA, thus affecting the treatment outcomes, therefore there is an urge to better understand OSA phenotypes, so pharyngeal surgery could be indicated aiming for the best outcome possible. There is a lack of a protocol relating objective findings with successful predictors for palatal surgery.

As suggested by the literature, the most effective predictors of surgical treatment in patients with OSA are BMI, Friedman classification and upper airway anatomy, the distance between the uvula and posterior wall amongst other cephalometry findings, AHI, positional OSA, level of obstruction in muller maneuver and DISE, and collapse improvement after placing the nasopharyngeal tube in DISE or during PSG.

### 4.1. BMI

It was shown by Shie et al. [25] and Millman et al. [20] that patients with OSA and a BMI greater than 27 kg/m^2^ have less of a chance of achieving success in surgical treatment. In another study by Zhang et al. in 2022 [36], it was stated that patients with a BMI greater than 27.5 kg/m^2^ have a worse response to the surgery compared to patients with a lower BMI. These observations impact the management of OSA colossally, since obesity is a very well-known comorbidity that can worsen OSA. Furthermore, OSAS can also cause prejudicial effects on cardiovascular conditions and comorbidities, and even worsen obesity. Therefore, it is essential that patients with obesity be treated correctly before and after the surgical approach to achieve a better prognosis.

### 4.2. Friedman

The Friedman tongue position (FTP) score, also known as the Friedman Scale, was developed in 1999 by Michael Friedman to describe and classify the morphology of the oropharynx with the tongue in a natural relaxed position [37]. Since OSAS is related to obstruction of the upper airway, it is crucial to know the point of obstruction of the upper airway in order to correct it. The Friedman score has been used to assess oropharynx obstruction, tonsil size, the Modified Mallampati (MM) classification, and the BMI, varying between 1 and 4.

It is already defined by its prognostic value in the literature, where the higher the Friedman score, the lower the chance of success of pharyngoplasty surgery. Ji Ho Choi et al. [38] and Michael Friedman et al. [28] reaffirm that in their study, the Friedman Scale I is a strong predictor of success in the uvulopalatopharyngoplasty on the other hand, Friedman Scale III and low hyoid position predicts surgical failure. Yousuf et al. in their article with 50 patients and a 92.2% success rate, agree that the Friedman score is an important predictor [35].

### 4.3. Cephalometry

Cephalometry is used to evaluate cranial proportions and angles. It is used in patients with OSA as a complementary exam to see whether the patient has a facial structure that constitutes a risk for developing or aggravated OSA. In some cases, the treatment of OSAS includes other surgeries other than pharyngoplasty, such as maxilomandibular advancement and glossectomy, to increase upper airway dimensions. Missale et al. [17] stated in their study that an anteroposterior pharyngeal distance greater than 8.5 mm was associated with low AHI and that anterolateral collapse may have some value in predicting value to pharyngoplasty surgery success. Liu et al. [19] showed that the distance between the posterior border of the uvula to the middle pharyngeal wall (U-MPW) was significantly longer in the UPPP responder group than in the non-responder group, taking patients with a U-MPW distance > 10 mm as a cutoff.

However, Doghramji et al. [26] showed in their study that cephalometric values cannot be used as a predictor of success in surgical intervention on patients with OSA, and were not able to properly identify surgical responders.

Due to conflicting literature, more studies are needed to truly define whether cephalometry can be a good predictor of success in pharyngeal surgery by itself.

A recent publication suggests the importance of the transverse dimension of the maxilla for predicting the tongue base obstruction and circumferential collapse at the velopharynx [39].

### 4.4. AHI

The apnea-hypopnea Index (AHI) is a parameter in polysomnography used to diagnose OSA. Apnea is a 10 s pause in breathing or more while you’re asleep. Meanwhile, hypopnea is a partial loss of breath for 10 s or longer. Few studies showed that the lower the AHI, the better the results after pharyngoplasty. According to Braga et al. [16] and Millman et al. [20], there is a correlation between AHI severity and the success of pharyngeal surgery, Millman went even further, classifying the patients on his study using an AHI of 38 as a cutoff, showing that patients with AHI lower than 38 have a better chance of success.

### 4.5. Level and Type of Obstruction at Muller Maneuver

The Muller maneuver is produced by a forced inspiration against a closed glottis, it is used to determine the level of obstruction on OSA while doing the nasopharyngolaryngoscopy, according to the VOTE classification (Velum, Oropharynx, Tongue base, and Epiglottis) [40] According to Yousuf et al. [35], patients with a positive Muller maneuver with palatal obstruction showed better results going through pharyngeal surgery. Missale et al. [17] also pointed out in their study that palatal obstruction and pharyngeal lateral collapse may be some good criteria to predict the success of pharyngeal surgery.

On the other hand, Doghramji et al. [26] discovered that the Muller maneuver is not a good parameter, since it was not able to distinguish between responders and non-responders of pharyngeal surgery.

Therefore, more research is needed to validate the capacity of using this parameter to predict surgical intervention success.

### 4.6. Positional Osa

Another predictor found in our research was positional OSA. According to Li et al. [41], in their study comparing patients with positional OSA to nonpositional OSA, the group with nonpositional OSA had higher AHI and Epworth Sleepiness scores, and the group with positional OSA had a significantly higher success rate after pharyngeal surgery, suggesting that this could also be used as a success predictor.

Further research is needed to evaluate whether positional apnea can be used as a reliable predictor of success in pharyngoplasty.

### 4.7. Drug-Induced Sleep Endoscopy (DISE)

Drug-induced sleep endoscopy (DISE) is a diagnostic tool that assesses the upper airway of snorers and OSA patients in dynamic conditions that mimic natural sleep. First described by Croft and Pringle in 1991 [42] DISE allows the physician to examine the sites of upper airway collapse which can be qualified and quantified with VOTE or NOHL classifications. The use of DISE aims to choose the best option to treat OSA [43]. A meta-analysis by Certal et al. found that surgical planning based on awake examination could change up to 50% of cases after DISE [44]. Thus, DISE allows personalized surgical planning and improves the success rate [45]. However, most of the series report results of multiple-level surgery and do not focus on pharyngeal surgery alone, making it difficult to interpret results. Zhang P. et al. [46] performed a study to see if the obstruction length (defined as the distance from the most superior point of the collapse to the most inferior point of the collapse) and obstruction height (the distance from the posterior border of the nasal septum to the most proximal point of the collapse), measured by both DISE and a pressure transducer catheter method were good predictors for success in velopharyngeal surgery. Through this study, they defined that an obstruction length > 1.4 cm and an obstruction height ≥ 3.2 cm were the only independent predictors of velopharyngeal surgery success.

There was only this study mentioning DISE to measure the distances used as parameters and we need to take into consideration that the values are dependent operators, meaning that it can be biased depending on who measures. Nonetheless, there was an important correlation between these values and surgery success in their study, so more research is needed to confirm this data.

In a multicentric retrospective study on 257 adult patients without tonsillar hypertrophy who underwent pharyngeal surgery for OSA, Green et al. [47]. studied the association between DISE findings and surgical outcomes on AHI. 97 patients (35%) underwent isolated palatal surgery after DISE, and the outcomes were not associated with VOTE findings.

Koutsourelakis et al. [48]. reported in a retrospective series of 49 patients who underwent palatal surgery with or without multilevel surgery, a higher proportion of circumferential velum complete collapse in non-respondents (42.3% vs. 4.3%).

A multicentric prospective non-randomized study led by Pang et al. [49] on 326 patients. 170 patients underwent DISE before surgery and were compared with the 156 non-DISE patients. There was no difference in outcomes between the two groups, but there was no subgroup analysis regarding palatal surgery alone and a bias due to the variation of practice between the institutions. A lateral oropharyngeal wall collapse was associated with higher surgical management failure rates in several studies [50,51].

Carrasco-Llatas et al. compared the results of different palatal surgery in 53 patients with OSA [52] after DISE selection. The surgical technique evolved over time with the aim to improve surgical outcomes, from partial palate resection, UPPP, and Z pharyngoplasty to lateral pharyngoplasty and expansion pharyngoplasty. They did not find any statistical difference regarding AHI outcomes according to the technique used, with a mean success rate of 71.7% according to Sher’s criteria.

Two studies did not find differences in UPPP outcomes in patients with [53] isolated pharyngeal obstruction versus multilevel DISE collapse patterns.

### 4.8. Nasopharyngeal Tube

Qiao et al. [54] compared the diagnosis of retrolingual obstruction using DISE and nasopharyngeal tube polysomnography, with an extent agreement of 80.6% between the two groups. It suggested that PSG with a nasopharyngeal tube may be reliable in diagnosing retrolingual obstruction when DISE is not available.

Victores et al. [55] suggested the use of nasopharyngeal tubes during DISE to predict the success of palatal surgery, this is useful as increased Pcrit (collapsible upper airways), may have more multilevel collapse before velopharyngeal surgery due to increased air resistance. Decreasing nasal and velopharyngeal turbulent airflow, in certain patients, may decrease or eliminate lower collapses. Li et al. [56] conducted sleep monitoring before and after nasopharyngeal tube placement to select patients for surgery without DISE, when AHI remained >15 using the tube. They hypothesize that if the nasopharyngeal tube was not enough efficient, this would translate to the presence of inferior obstruction. Thus, they proposed UPPP combined with glossopharyngeal surgery according to patients. Of 59 patients, 34 accepted the multilevel surgery, and 25 refused glossopharyngeal surgery and received only UPPP. The success rates of surgery were 82.3% and 40.0% respectively, suggesting the failure of the nasopharyngeal tube to improve AHI, which suggests multilevel collapse, explaining the success rate of UPPP alone.

Dellweg et al. [57] assessed the use of a nasopharyngeal tube to reduce palatal collapse, inserted in the first half of the night, compared to the second half of the night without the tube. They used the wrist-worn WatchPat^®^ device to measure AHI and performed DISE with and without the nasopharyngeal tube the next morning. Of the 50 patients presenting with palatal obstruction at DISE, 41 (85.7%) were diagnosed at night when the threshold reduction of AHI was set at 40%.

## 5. Conclusions

The lack of an accurate protocol for the indication of pharyngeal surgery is a reality, however, with some phenotypes, it is possible to predict the success of the procedure. In this narrative review of adult OSA, the main types of factors of success identified as OSA pharyngeal surgery responders are explored. Information alluding to predictors of success may be obtained from the physical examination and diagnostic tools. The former includes a BMI < 27 kg/m^2^, a Friedman scale 1, and isolated pharyngeal obstruction during the muller maneuver (controversial). The latter, cephalometry with >8.5–10 mm retropalatal distance and increased transverse maxilla dimension, isolated pharyngeal obstruction on DISE (controversial), DISE with a pressure transducer catheter length > 1.4 cm and obstruction height ≥ 3.2 cm, DISE or PSG with a decrease in the AHI after nasopharyngeal tube placement, or PSG data revealing an AHI < 38/h and/or positional OSA. Further studies may help to better understand the intersection between phenotypes and success predictors of pharyngeal surgery.

## Figures and Tables

**Table 1:** Clinical data of articles included in the review.

Title	Year of Publication	Predictor for Success	Number of Patients	Success Rate
Predictors of uvulopalatopharyngoplasty success in the treatment of obstructive sleep apnea syndrome [16]	2013	BMI, AHI, Cephalometry	54	-
Outcome predictors for non-resective pharyngoplasty alone or as a part of multilevel surgery, in obstructive sleep apnea-hypopnea syndrome [17].	2020	AHI, Cephalometry	70	-
Predictors of surgical outcomes of uvulopalatopharyngoplasty for obstructive sleep apnea hypopnea syndrome [18].	2011	AHI	39	56.4%
Associated predictors of therapeutic response to uvulopharyngopalatoplasty for severe obstructive sleep apnea hypopnea syndrome [19].	2013	AHI, Cephalometry	51	45.1%
Simple predictors of uvulopalatopharyngoplasty outcome in the treatment of obstructive sleep apnea [20].	2000	BMI, AHI, Cephalometry	46	-
Five-Year Objective and Subjective Outcomes of Velopharyngeal Surgery for Patients with Obstructive Sleep Apnea [21].	2019	Tonsil Size and ODI	63	66.6%
Use of morphological indicators to predict outcomes of palatopharyngeal surgery in patients with obstructive sleep apnea [22].	2004	BMI, Tonsil Size, Mallampati, AHI	105	-
The Combination of Anatomy and Genioglossus Activity in Predicting the Outcomes of Velopharyngeal Surgery [23].	2017	Cephalometry, Genioglossus Electromyography	40	62.5%
The Role of Genioglossus Activity in Predicting Uvulopalatopharyngoplasty Outcomes [24].	2019	Tonsil Size, Genioglossus Electromyography	44	92.9%
Impact of obesity on uvulopalatopharyngoplasty success in patients with severe obstructive sleep apnea: a retrospective single-center study in Taiwan [25].	2012	BMI, AHI, Friedman, Epworth	117	24.6%
Predictors of outcome for uvulopalatopharyngoplasty [26]	1995	Fiberoptic nasopharyngolaryngoscopy with the Müller maneuver (FNMM), Cephalometry	53	32.1%
Clinical staging for sleep-disordered breathing [27].	2016	BMI, Tonsil Size, Palate Position at Cephalometry	134	80.6%
Does severity of obstructive sleep apnea/hypopnea syndrome predict uvulopalatopharyngoplasty outcome? [28]	2009	AHI, Friedman	134	31.3%
Obstructive sleep apnea syndrome: preoperative radiologic evaluation. [29]	1988	Palate Position with Cephalometry	12	-
Mouth opening during sleep may be a critical predictor of surgical outcome after uvulopalatopharyngoplasty for obstructive sleep apnea [30].	2010	Oral Cavity Anatomy	69	-
Upper airway anatomical changes after velopharyngeal surgery in obstructive sleep apnea patients with small tonsils [31].	2009	Tonsil Size, Cephalometry	44	72.4%
Body mass index less than 28 kg/m^2^ is a predictor of subjective improvement after laser-assisted uvulopalatoplasty for snoring [32]	2009	BMI	119	-
Is there a relationship between tonsil volume and the success of pharyngeal surgery among adult patients with obstructive sleep apnea? [33]	2022	Tonsil Size	44	65.6%
Efficacy, predictors of success and failure of an updated lateral pharyngoplasty approach as an independent procedure in treating obstructive sleep apnea with CPAP failures [34]	2021	Type of collapse in DISE	46	69.9%
Clinical predictors for successful uvulopalatopharyngoplasty in the management of obstructive sleep apnea [35]	2013	BMI, Friedman, Muller Maneuver, Neck Circumference	50	95.2%

## Data Availability

Not applicable.
